# Pulmonary embolism due to hemangioma of segment I compressing the inferior vena cava: A Case Report

**DOI:** 10.1016/j.ijscr.2020.07.038

**Published:** 2020-07-15

**Authors:** Sarah Winterland, Tim Reese, Georgios Makridis, Karl J. Oldhafer

**Affiliations:** a Department of Surgery, Division of Hepatobiliary and Pancreatic Surgery, Asklepios Hospital Barmbek, Hamburg, Germany; b University Hospital Hamburg Eppendorf, Hamburg, Germany; c Semmelweis University of Medicine Budapest, Asklepios Campus Hamburg, Hamburg, Germany

**Keywords:** Surgery, Liver resection, Symptomatic hepatic hemangioma, Pulmonary embolism, Compression of the vena cava

## Abstract

**Introduction:**

Hemangiomas are common hepatic lesions and are mostly asymptomatic. Operative removal should only be performed when the lesions are symptomatic, and removal of the hemangioma is deemed to be more beneficial than the risk of the operation itself.

**Presentation of the case:**

The multiple hemangiomas of our patient were discovered nine years before first symptoms. The patient presented with pulmonary artery embolism due to a compression of the vena cava inferior by a hemangioma in segment I. No other cause of thromboembolic events could be diagnosed. We performed surgical enucleation of the hemangioma, which resulted in the normalization of blood flow in the inferior vena cava. As the inducing factor for clot development was removed, no permanent thrombosis prophylaxis was implicated.

**Discussion:**

Pulmonary embolisms owing to compression of the inferior vena cave are rare.

**Conclusion:**

Until now to our knowledge, pulmonary embolisms have never been described as an indication for hepatic hemangioma enucleation.

## Highlights

• Compression of the Vena cava leading to pulmonary embolism is a rare symptom of hemangioma.
• Resection of Hemangioma to normalize the flow of the vena cava is a rare indication, but an effective and safe therapy.
• The patient required no anticoagulation after the surgery.

## 1 Introduction

Hepatic hemangiomas (HH) are the most common type of benign liver tumors with a prevalence of up to 20% [1]. About 50–70% of the cases are described to be asymptomatic [2]. Often, HH is an incidental finding in radiological imaging [3]. Most common symptoms caused by pressure on adjacent structures are unspecific and include abdominal pain, nausea and jaundice [3,4]. As an operation itself poses a risk, a careful evaluation of the hemangioma has to be conducted using modern imaging to weigh the risk against the benefit of tumor removal [5]. Therefore, a decision should be made on a case to case basis and consider the patients history, other probable explanations for the symptoms and expected benefit because in 25% of the cases alleviating all symptoms cannot be achieved by tumor removal [1]. In this report, we present a case with a segment I HH causing thromboembolic complications including pulmonary artery embolism treated by HH enucleation. This case report has been reported in line with the SCARE criteria [6].

## 2 Clinical case

**Fig. 1:**
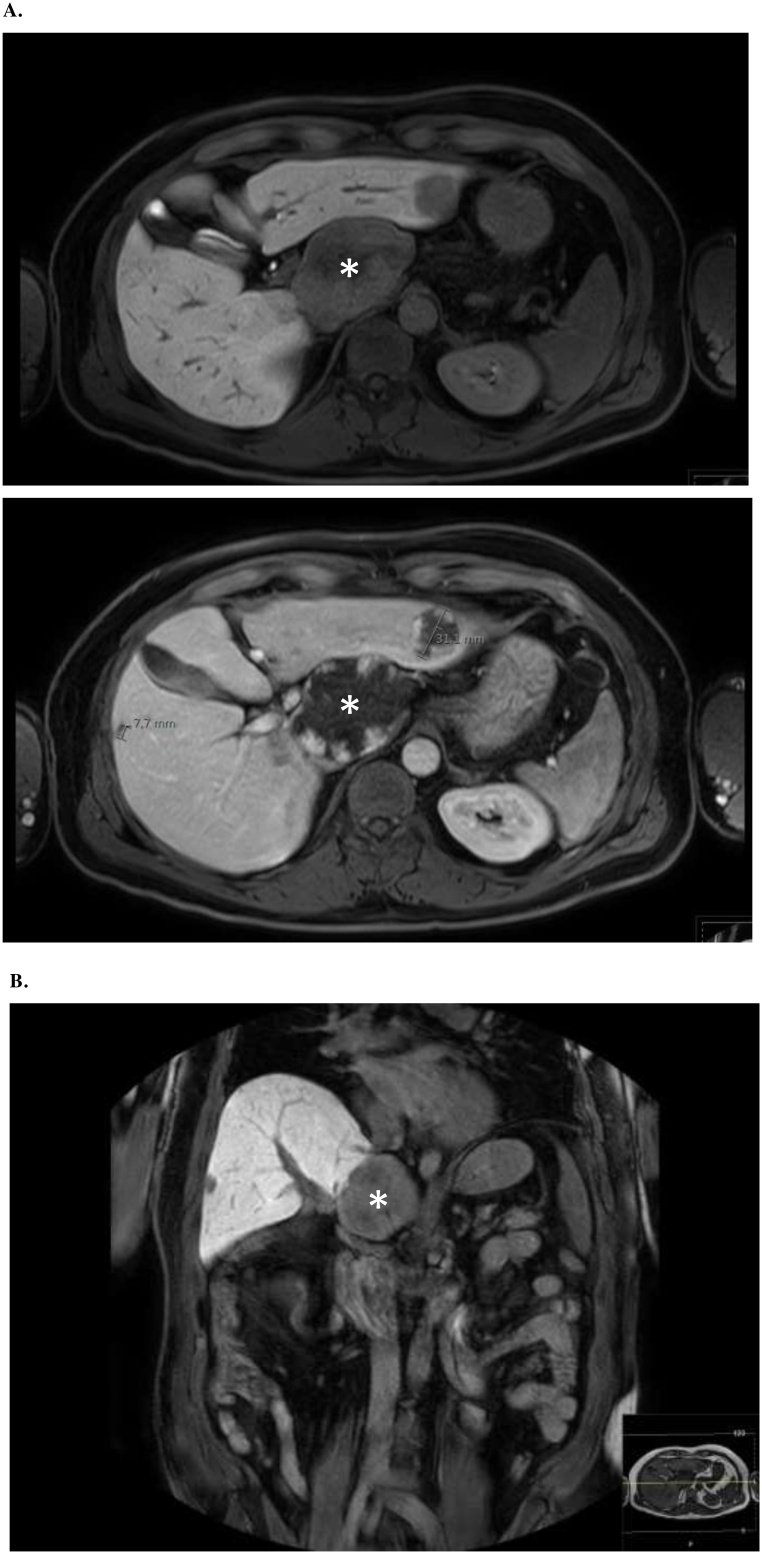
MRI images showing multiple hemangiomas of the liver. The largest HH is located in segment I (*) compressing the inferior vena cava. A: Axial section MRI. B: Coronal section MRI.

**Fig. 2:**
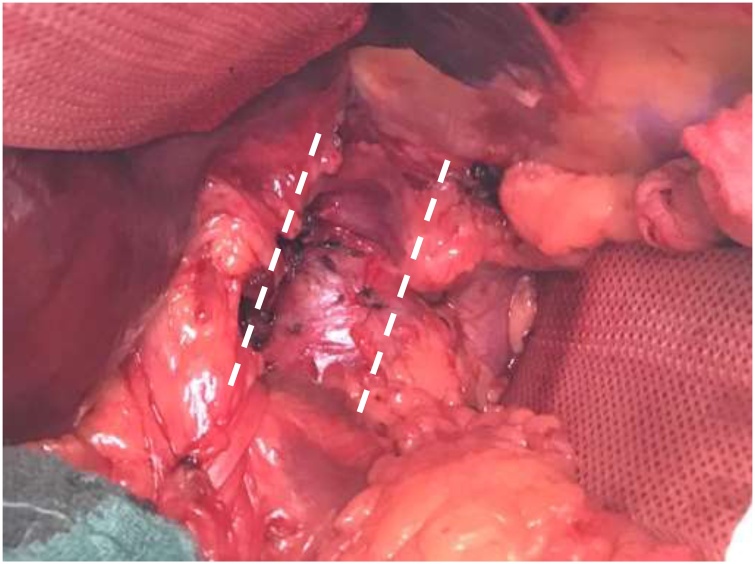
Intraoperative situs after removal of the hemangioma. The lines indicate the vena cava.

**Fig. 3:**
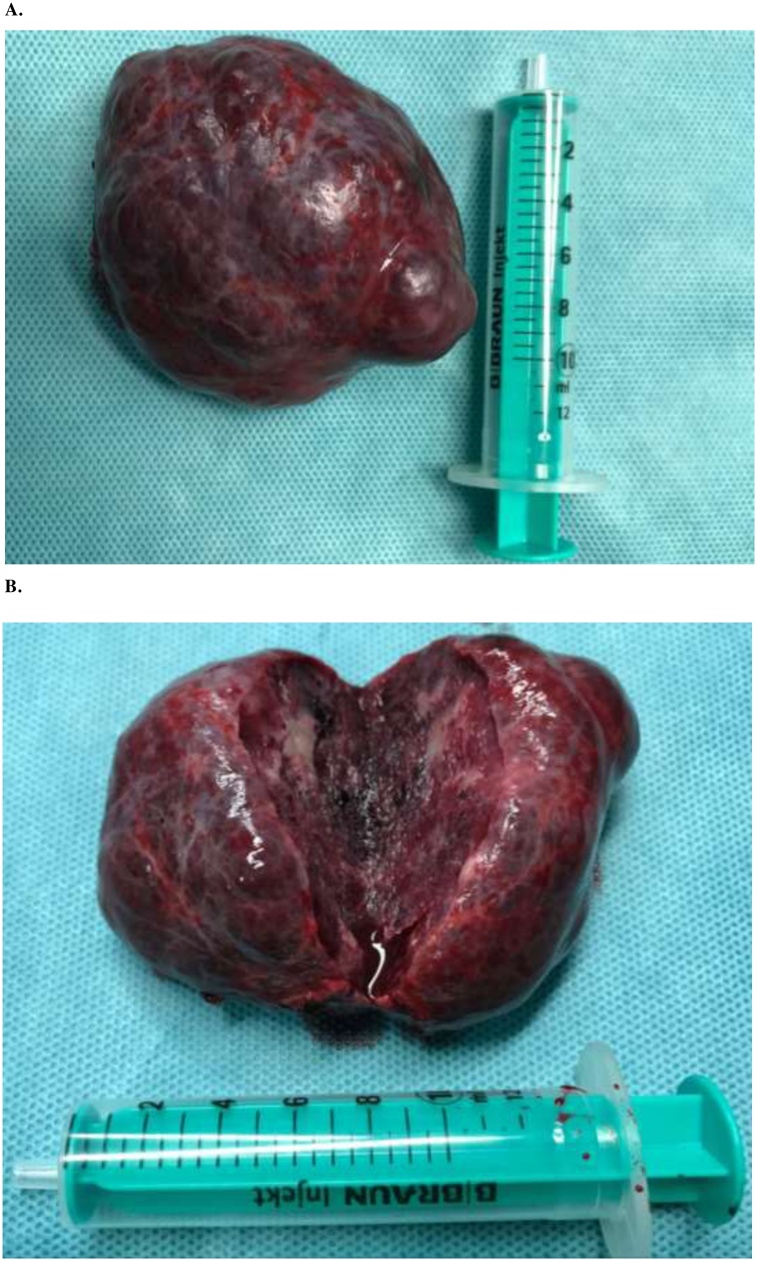
Top view on the hemangioma after operative removal. 10 mL syringe as size comparison. A: Hematoma in its entirety B: Crosssection.

A 50-year-old male presented after having suffered a pulmonary artery embolism four month earlier. A magnetic resonance tomography (MRI) showed multiple HH. Those were first discovered incidentally nine years prior and were now growing in size. The patient had not experienced local symptoms related to the HH like pain or nausea. The largest HH measured 83 × 57 mm (Fig. 1A+B) and was located in the segment I compressing the inferior vena cava (IVC). Three months before the hemangioma measured only 73 × 51 mm. Furthermore, the patients` history revealed two episodes of deep vein thrombosis (DVT). An underlying coagulopathy could not be diagnosed. The patient was a non-smoker who also suffered from type 2 diabetes mellitus, sarcoidosis of the lung and rheumatoid arthritis treated with methotrexate. Therefore, the HH compressing the IVC was presumed to cause the DVT and pulmonary embolism due to hemostasis proximal of the IVC. To reduce the risk for recurrent DVT or pulmonary embolisms, we decided to remove the symptomatic HH. Liver biochemistry on admission was normal. An intraoperative ultrasound showed a pathologic, accelerated flow pattern in the IVC caused by the tumor compression in segment I. Enucleation of the HH in segment I was performed in an open approach (Fig. 2). After removal (Fig. 2) no residual compression was observed and a normal venous flow pattern of the IVC was detected with duplex ultrasound. The HH (Fig. 3A+B) appeared to be highly vascularized and showed bleeding on contact. The remaining HH were presumed to be asymptomatic and were therefore not removed. Final pathology confirmed the HH with no signs of malignancy. The postoperative course was uneventful and the patient was discharged 6 days after surgery. The anticoagulation for thrombosis prevention was stopped after discharge. A sonography three months after discharge showed a normal blood flow in the IVC.

## 3 Discussion

Hemangiomas are benign hamartomatous tumors, with a prevalence of 1–20%, that rarely show symptoms [1,2]. The tumors are more likely to appear in women (female:male ratio = 5:1) and are most often discovered incidentally between 30 and 50 years of age [5]. Growth of the lesions may occur during pregnancy [7] and an influence of oral contraceptive remains controversial [5]. Lesions bigger than 5 cm in diameter are considered giant hemangiomas [5,8]. These lesions are more frequently symptomatic than smaller lesions [5]. Due to their size HH can also cause compression of major intraabdominal structures [9]. Case reports demonstrate the possible compression of the bile duct, the hepatic veins, the portal vein and the IVC [4,9]. Predeterminating factors for the symptoms and complications are size, growth rate and localization in the liver [9].

Compression of the IVC is described in two case reports and resulted in lower limb edema as described by Akbulut et al. [9] and in pulmonary embolism as described in 1993 by Paolillo et al. [4]. The compression of the IVC led to hemostasis which was the cause of the thromboembolic event. Paolillo et al. could not achieve an operative removal of the tumor as the hemangioma showed multisegmental and perihilar involvement [4]. In our case, the HH could be removed by enucleation. A clear benefit of removal was to be expected as the pulmonary artery embolism was presumed to be a direct cause of the size of the HH and its resulting compression of the IVC. Because the HH could be removed by simple enucleation and a complete resection of segment I seemed not be necessary an operative approach was deemed to be more beneficial than an embolization as it resulted in the immediate removal of the compression. The interventional placement of an IVC stent was also discussed as an alternative treatment option [10]. However, literature on venous stenting of IVC compression by tumors and own institute`s experience are relatively limited. Therefore, IVC stenting was discarded.

In conclusion, in selected cases of IVC compression owing to benign liver tumors leading to pulmonary embolism tumor enucleation/resection seems to be an effective and safe therapy. Thus, beside severe local complications, symptomatic IVC compression represents a seldom but possible indication for surgical therapy in HH patients.

## Declaration of Competing Interest

Nothing to declare.

## Funding

Nothing to declare.

## Ethical approval

No ethical approval was required for this case report.

## Consent

Patient gave consent for publishing.

## Author contribution

Sarah Winterland wrote the manuscript.

Tim Reese wrote the manuscript.

Gorgios Makridis critically reviewed the manuscript.

Karl J. Oldhafer critically reviewed the manuscript.

## Registration of research studies

N/A.

## Guarantor

Tim Reese.

Karl J. Oldhafer.

## Provenance and peer review

Not commissioned, externally peer-reviewed.
